# Gall Bladder Agenesis: An Incidental Intraoperative Finding in a Case of Stab Injury

**DOI:** 10.7759/cureus.84200

**Published:** 2025-05-15

**Authors:** Rashmi Sahu, Abdul Haque M Quraishi, Kishan Kumar Meena, Girish Umare

**Affiliations:** 1 General Surgery, Government Medical College & Hospital, Nagpur, IND; 2 Surgery, Government Medical College & Hospital, Nagpur, IND

**Keywords:** exploratory laparotomy, gall bladder agenesis, incidental finding, magnetic resonance cholangiopancreatography, ultrasonography

## Abstract

Agenesis of the gall bladder is extremely rare. Three types of gall bladder agenesis have been described. The asymptomatic type is diagnosed incidentally on imaging, intraoperatively, or at autopsy. Symptomatic patients present with clinical features such as biliary colic, usually in the 4th and 5th decades. The third type presents in neonatal life along with multiple fetal anomalies.

We present a case of a 43-year-old male who presented to our trauma casualty with a history of multiple stab injuries to the abdomen. There was no history of previous abdominal surgery. An ultrasonography and computed tomography of the abdomen showed the absence of the gall bladder. Intraoperatively, there were multiple, through-and-through bowel perforations with no evidence of other organ injury. The gall bladder and cystic duct were absent, and the same was confirmed on a post-operative magnetic resonance cholangiopancreatography.

This article emphasizes the importance of seeking and documenting incidental anatomical anomalies such as gall bladder agenesis to avoid difficulties in diagnosis if related symptoms arise in the future.

## Introduction

The gall bladder develops from the cystic yolk during the fourth week and the cystic duct during the seventh week of intrauterine life. The pathogenesis of gall bladder agenesis is unclear. There are two proposed theories regarding this [1]. The first suggests that a failure of the development of the hepatic diverticulum bud of the foregut leads to malformation. The second one proposes an inaccuracy in the process of canalization of the cystic duct and gallbladder. The hepatic diverticulum forms the liver, and its connection to the gut narrows and forms the extrahepatic biliary system. An outgrowth from this narrowed segment canalizes and forms the gall bladder and cystic duct. When this outgrowth fails to develop, it leads to agenesis of the gall bladder without affecting the normal development of the extrahepatic biliary system [2].

Developmental abnormalities of the gall bladder are relatively uncommon. Gall bladder agenesis is an extremely rare congenital anomaly with an incidence of 10-65 per 1,00,000. It is three times more common in females than males [3]. Most cases are asymptomatic; diagnosis is an incidental finding on imaging, intraoperatively, or at autopsy [4].

We present an intraoperative finding of gall bladder agenesis during exploratory laparotomy in a stab injury over the abdomen with no previous operative history. According to classification by Bennion et al. [2] and Tang et al. [5], our case belongs to the asymptomatic type. The case report emphasizes the need of documenting anomalies noted incidentally to avoid diagnostic problems in case of symptoms in the future.

## Case presentation

A 43-year-old male presented to the trauma casualty with a history of multiple stab injuries over the abdomen sustained before 4-5 hours of presentation. He had no previous operative history. He had been consuming alcohol for 13 years. The patient was drowsy on examination, with a pulse rate of 110 beats per minute, blood pressure of 100/60 mm Hg, and an inability to maintain oxygen saturation on room air. On examination, there were two stab wounds on the anterior chest wall and four stab wounds on the abdomen. On palpation, abdominal guarding was present, with three out of four abdominal stab wounds communicating with the peritoneal cavity.

**Figure 1:**
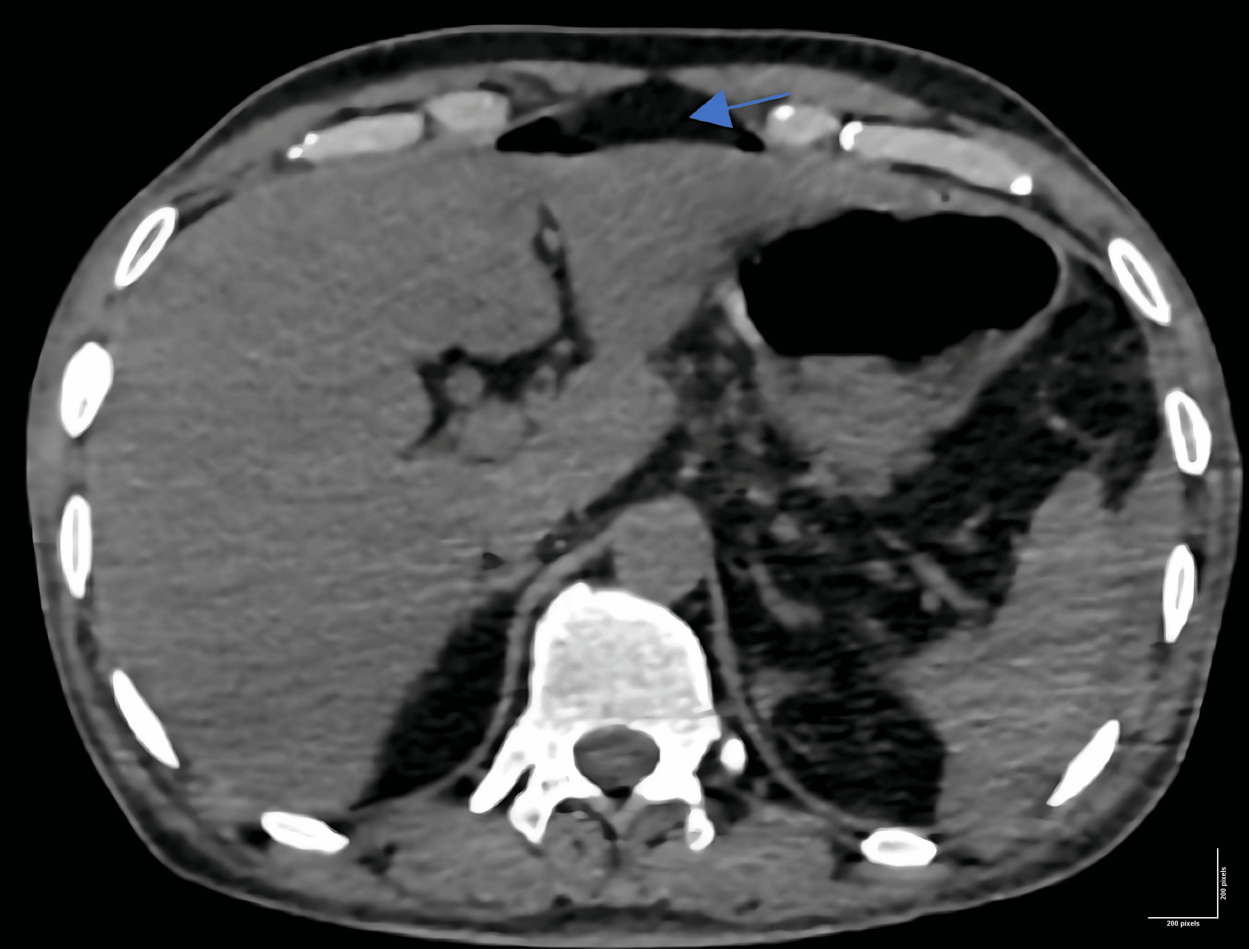
CT scan shows pneumoperitoneum (arrow) with an absent gall bladder.

On USG of the abdomen, there was no evidence of intraperitoneal collection or solid organ injury. The gall bladder was not visualized. Pre-operatively, the patient was considered for non-contrast CT of the thorax, abdomen, and pelvis. It suggested the presence of bilateral mild hemothorax and pneumoperitoneum (Figure 1), and the gall bladder was not visualized even on CT. Blood investigations were performed, including a complete blood count and renal and hepatic functions, all of which were within the normal range of reference.

**Figure 2:**
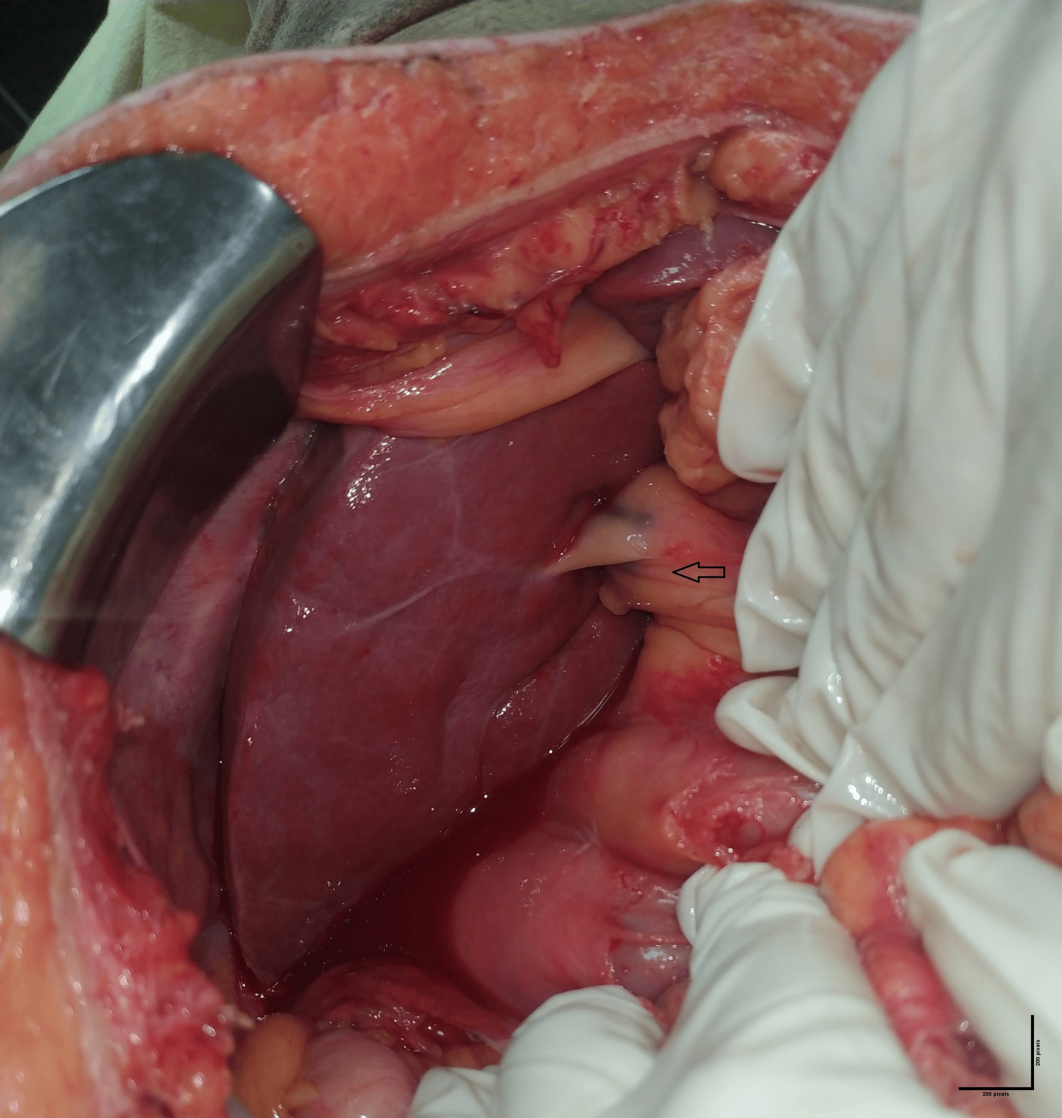
The absence of the gall bladder and cystic duct intraoperatively. The common bile duct (CBD) enters the liver. The arrow represents CBD.

A provisional diagnosis of perforation peritonitis secondary to stab injury was made, and the patient underwent emergency exploratory laparotomy after resuscitation. On exploration, there was no evidence of hemoperitoneum or intraperitoneal collection. During laparotomy, the small bowel was found to have a through-and-through perforation of size 0.5 x 0.5 cm at a distance of 60 cm, 140 cm, and 150 cm from the duodenojejunal junction. Another perforation was found at the hepatic flexure of size approximately 1 x 1 cm. All the perforations were repaired in two layers with Vicryl (polyglactin) 3-0 and silk 3-0 round body after a thorough peritoneal wash with normal saline. Spleen and liver were identified with no evidence of injury, but the gall bladder was not identified in its anatomical site (Figure 2).

**Figure 3:**
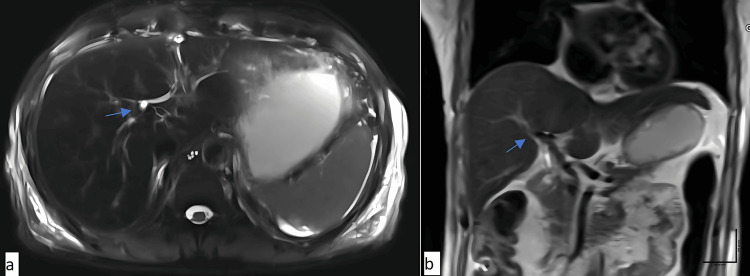
Post-operative magnetic resonance cholangiography: (a) axial and (b) coronal view. Agenesis of the gall bladder is indicated by an arrow.

**Figure 4:**
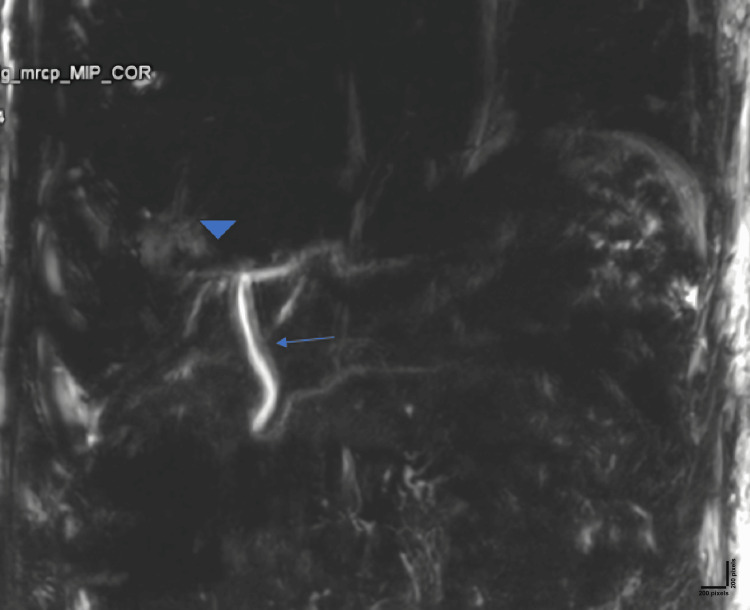
Post-operative magnetic resonance cholangiography shows agenesis of the gall bladder and cystic duct (arrowhead) and common bile duct (arrow).

Postoperatively, the patient was considered for an magnetic resonance cholangiopancreatography (MRCP) scan. It confirmed agenesis of the gall bladder and cystic duct (Figures 3, 4). Intrahepatic biliary radicals were normal with no evidence of dilation. Common hepatic duct measured 4.4 mm with normal course and caliber. The common bile duct measured 4.6 mm with no intraluminal filling defect. The post-operative period was uneventful, and the patient was discharged from hospital after one week. During follow up he was asymptomatic.

## Discussion

We have presented here a case of gall bladder agenesis, which was found incidentally intraoperatively during an exploratory laparotomy for a case of abdominal stab injury. This is a unique presentation of a rare anomaly of the gall bladder. Discovery of this anomaly during exploratory laparotomy performed for an unrelated cause (penetrating trauma) was not found to be reported in the literature during our search.

Gallbladder agenesis was first reported in the medical literature by Lemery in 1701 and Bergman in 1702 [3,6]. It is rare with an incidence of 10-65 per 1,00,000 [7,8] but based on autopsy, the incidence increased to 90 per 1,00,000 [9]. In 1988, Bennion et al classified three groups based on presentation in gall bladder agenesis [2]. An asymptomatic group (35%) was the first group where gall bladder agenesis was found incidentally on imaging, at laparotomy for unrelated diagnosis, or autopsy. The second was a symptomatic group (50%) presenting in the 4th or 5th decade, usually as an isolated anomaly presenting with a biliary colic. The third group was of neonates with multiple fetal anomalies (15%). A study by Tang et al. in 2015 proposed a new classification that divides gall bladder agenesis into life-threatening and non-life-threatening conditions. It also corrects the overlap that exists in Bennion's classification between the first and third groups. They have described a symptomatic type that can be further divided into Ia and Ib subtypes. Type Ia is accompanied by lethal deformities such as biliary atresia, ventricular septal defect, imperforate anus, duodenal atresia, etc. (most of them dying shortly after birth) and type Ib is accompanied by non-lethal malformations such as intestinal malrotation, right liver agenesis, cryptorchism, choledochal cyst, and choledochectasia. Type II is the asymptomatic type [5,10].

Symptomatic patients present with complaints similar to biliary colic in gall bladder agenesis, but the mechanisms are not known. Most authors consider it to be due to dysfunction of the sphincter of Oddi and biliary dyskinesis [1]. Abdominal ultrasound is the initial investigation when there is suspicion of biliary pathology because of its high sensitivity in diagnosing gallstones [11]. In gallbladder agenesis, false positive results are most frequently reported as a shrunken, contracted, or constricted gallbladder with hyperechoic shadowing suggestive of gallstones [12]. This is likely to result in avoidable surgical intervention. There have been cases reported where a laparoscopic cholecystectomy was performed, and on finding an absent gall bladder, the procedure was converted to open. The diagnosis was finally confirmed on a post-operative MRCP [13].

Our case emphasizes the need to seek and note such incidental intraoperative findings, which pose diagnostic challenges. It also stresses the need for doing a post-operative MRCP to confirm the same. This prevents post-operative misdiagnosis and unnecessary interventions, should the patient present with related symptoms. Akin to agenesis of the gall bladder, there are other anomalies such as duplication of the gall bladder, which can present diagnostic issues. In the latter case, knowledge of various types of duplication is important from the management's point of view [14].

MRCP is a non-invasive modality that provides excellent images of the gall bladder and biliary system. Even if the agenesis is diagnosed intraoperatively, MRCP should be considered postoperatively to exclude an ectopic gallbladder; most common sites of which are intrahepatic, retrohepatic, within the leaves of lesser omentum or falciform, and retroperitoneum [15].

Intraoperative ultrasonography could have been performed in our case to search for a possible ectopic gall bladder. However, due to the unavailability of the same, it was not performed. In any case, extending operative time to look for a probable anomaly in the face of an evident emergency is also debatable.

Our case is unique, as a rare anomaly of the gall bladder has been found in an unexpected clinical situation. The report highlights the importance of noting such incidental intraoperatively found anomalies and following it up with investigations to confirm the diagnosis. Awareness of this will prevent post-operative diagnostic problems in future and help in making correct decisions in management.

## Conclusions

This unique case of gall bladder agenesis found incidentally during abdominal exploration in a case of trauma emphasizes the need to find and document the anomaly found intraoperatively and further investigate the same by a post-operative MRCP for confirmation. This exercise is meant to rule out gall bladder pathology as a cause of abdominal pain in the future. The USG of the abdomen can produce a false positive report of cholelithiasis in this anomaly, leading to an avoidable abdominal exploration. Awareness of this condition avoids an embarrassing situation for the surgeon.
